# Where Does Yarigai Come From? Meaning and Fulfilment in Medicine When Cure Is Limited

**DOI:** 10.1111/medu.70242

**Published:** 2026-05-13

**Authors:** Hiroshi Nishigori

**Affiliations:** ^1^ Center for Medical Education Nagoya University Nagoya Japan

## Abstract

When cure is not possible, where do physicians find fulfilment? This commentary explores “yarigai” as a lens to rethink meaning in medicine and medical education. #MedEd #Geriatrics

In this issue, Draper and colleagues study medical students' experiences in caring for older patients through a qualitative approach incorporating rich pictures, offering a nuanced account of emotion, relationships and uncertainty in clinical practice.^1^ The findings suggest that caring for older patients is experienced not merely as the application of clinical skills, but as an encounter imbued with meaning and fulfilment. While such dimensions are not unique to geriatric care and may be inherent to medicine more broadly, they appear particularly visible within this context.

Viewed in this way, the study does more than describe a specific clinical domain. It provides a lens through which to reconsider a more fundamental question: Where does meaning in medicine arise, and what constitutes fulfilment for physicians?

Historically, medicine has developed around a problem‐solving logic: identifying disease, intervening appropriately and achieving improvement. Medical education has largely reflected this orientation as characterised by problem‐based and case‐based learning. Within such a framework, fulfilment is often closely tied to achievement. Diagnosing correctly, treating effectively and observing recovery have traditionally been seen as central sources of value and satisfaction in medical practice.

However, the clinical contexts depicted in this study challenge this assumption. Multimorbidity, chronic illness and end‐of‐life care often do not allow for clear improvement or cure‐related notions of ‘success’. In such situations, students may experience frustration, powerlessness and a sense that ‘nothing much happened’.^1^ In this sense, geriatric care may be perceived as a domain where fulfilment is harder to attain in conventional terms.^2^

Yet, the study also reveals that students do experience deep fulfilment within these very contexts. Importantly, this fulfilment does not arise from curing disease, but from being present, building relationships and contributing meaningfully to patients and their families.

To better understand this, it is helpful to introduce the Japanese concept of *yarigai*.^3^ Yarigai does not have a direct one‐word equivalent in English and is sometimes translated as fulfilment or a sense of worthwhileness, although such translations remain partial. Yarigai is not limited to outcomes or achievements; rather, it encompasses a sense of meaning experienced through engagement in work or action itself. It is also often associated with *ikigai*, a broader concept referring to what makes life worth living. Importantly, yarigai is not reducible to a single source.

Yarigai is not reducible to a single source.

From this perspective, the findings of this study suggest that the structure of yarigai becomes configured differently in the context of geriatric care. When clear outcomes are difficult to achieve, fulfilment derived from achievement does not disappear, but relational forms of meaning‐making become more salient. Medicine, in this sense, can be understood as encompassing both ‘achieving’ and ‘continuing to engage’.

Such a shift invites reconsideration of assumptions embedded in medical education. Many students enter medicine with a strong orientation toward cure, while care, particularly relational and supportive practices, may be perceived as secondary. In some cases, such aspects are implicitly positioned as belonging to other professions, such as nursing, reflecting a tacit division of labour.

At the same time, in ageing societies, the limits of cure are increasingly evident. In Japan—similar to the Netherlands, where this study was conducted, but with an even more rapidly ageing population—these challenges are equally, if not more, pronounced. The question of what physicians offer when cure is not possible becomes central. This is not only a clinical question, but one that speaks to the very meaning of medicine. Seen in this light, the contribution of Draper et al.'s work lies less in providing answers than in opening up important questions—questions that may be framed in terms of how yarigai is constituted in medical practice.

The question of what physicians offer when cure is not possible becomes central. … This is not only a clinical question, but one that speaks to the very meaning of medicine.

First, there is the question of physicians' motivation. If yarigai arises from multiple dimensions, such as achievement and relational engagement, what balance between these should physicians ideally maintain? Moreover, yarigai cannot be reduced solely to achievement or relationships; it remains unclear what other sources may give rise to it. There is also a need to reconsider the broader question of how physicians' yarigai may emerge from a diverse range of sources.

Second, there is the question of professional roles. If relational care becomes an important source of yarigai, how should responsibilities be redistributed across professions? In particular, how should physicians, especially geriatricians and family physicians, engage with aspects of care that have traditionally been associated with nursing and other health professionals? Furthermore, such engagement raises the question of whether this should be understood merely as an expansion of roles, or whether it calls for a redefinition of professional identity itself.

Third, there is the question of technological change. As advances such as artificial intelligence reshape diagnostic and therapeutic practices, how might the sources of yarigai shift? Will achievement‐based fulfilment diminish in relative importance, with relational dimensions becoming more central, or will new configurations emerge? Moreover, as certain aspects of medical work become increasingly automated, where will physicians locate meaning in their practice, and how might such sources of yarigai be reconstituted?

These questions remain open. The experiences described in this study suggest that yarigai is not a fixed construct, but a dynamic phenomenon—one that emerges, evolves and sometimes falters within specific clinical contexts.

Yarigai is not a fixed construct, but a dynamic phenomenon—one that emerges, evolves and sometimes falters within specific clinical contexts.

Future research is therefore needed to examine how yarigai among physicians develops and evolves. In particular, rather than assuming a limited set of sources, it will be important to explore how yarigai may emerge from a diverse and potentially shifting range of experiences, shaped by education, clinical practice, healthcare systems and technological change.

Geriatric care is sometimes perceived as a field in which little can be done. Yet this study suggests otherwise. Even in situations where cure is not possible, yarigai may still arise, not only through achievement or relational engagement, but through forms of meaning‐making that remain only partially understood. Yarigai, therefore, does not disappear in the face of uncertainty and limitation; rather, it may take on different forms that remain only partially understood.

Even in situations where cure is not possible, yarigai may still arise, not only through achievement or relational engagement, but through forms of meaning‐making that remain only partially understood.

## AUTHOR CONTRIBUTIONS

**Hiroshi Nishigori:** Conceptualization; writing—original draft; writing—review and editing.

## CONFLICT OF INTEREST STATEMENT

The author declares no conflict of interest.

## Data Availability

The data that support the findings of this study are available from the corresponding author upon reasonable request.

## References

[1] Draper EJ , de laCroix A , Meiboom AA , vanDijk N , Kusurkar RA , Smalbrugge M . Medical students' experiences in providing medical care to older patients: a rich picture study. Med Educ. 2026;60(8):908‐917. doi:10.1111/medu.70181 41578706 PMC13350398

[2] Samra R , Cox T , Gordon AL , Conroy SP , Lucassen MFG , Griffiths A . Factors related to medical students' and doctors' attitudes towards older patients: a systematic review. Age Ageing. 2017;46(6):911‐919. doi:10.1093/ageing/afx058 28472444 PMC5860378

[3] Nishigori H , Shimazono Y , Busari J , Dornan T . Exploring yarigai: the meaning of working as a physician in teaching medical professionalism. Med Teach. 2024;46(11):1486‐1493. doi:10.1080/0142159X.2024.2316227 38376459

